# Tissue-specific laser microdissection of the *Brassica napus* funiculus improves gene discovery and spatial identification of biological processes

**DOI:** 10.1093/jxb/erw179

**Published:** 2016-05-18

**Authors:** Ainsley C. Chan, Deirdre Khan, Ian J. Girard, Michael G. Becker, Jenna L. Millar, David Sytnik, Mark F. Belmonte

**Affiliations:** University of Manitoba, Department of Biological Sciences, 50 Sifton Road, Winnipeg, MB R3T 2N2, Canada

**Keywords:** *Brassica napus*, canola, development, funiculus, RNA-seq, seed, transcriptome.

## Abstract

The three primary tissue systems of the funiculus each undergo unique developmental programs to support the growth and development of the filial seed. To understand the underlying transcriptional mechanisms that orchestrate development of the funiculus at the globular embryonic stage of seed development, we used laser microdissection coupled with RNA-sequencing to produce a high-resolution dataset of the mRNAs present in the epidermis, cortex, and vasculature of the *Brassica napus* (canola) funiculus. We identified 7761 additional genes in these tissues compared with the whole funiculus organ alone using this technology. Differential expression and enrichment analyses were used to identify several biological processes associated with each tissue system. Our data show that cell wall modification and lipid metabolism are prominent in the epidermis, cell growth and modification occur in the cortex, and vascular tissue proliferation and differentiation occur in the central vascular strand. We provide further evidence that each of the three tissue systems of the globular stage funiculus are involved in specific biological processes that all co-ordinate to support seed development. The identification of genes and gene regulators responsible for tissue-specific developmental processes of the canola funiculus now serves as a valuable resource for seed improvement research.

## Highlight

Tissue-specific transcriptomic analysis reveals biological processes contributing to the development of the epidermis, cortex, and vasculature, and how these tissues contribute to the development and function of the canola funiculus.

## Introduction

Biological processes required to program development are controlled by large suites of genes that can be separated between tissues of the plant. The three tissue systems—dermal, ground, and vasculature—must undergo strictly co-ordinated development to serve their unique functions within a plant organ. The morphological diversity between and within plant tissue systems is indicative of the compartmentalization of biological function. For example, the epidermis functions primarily in protection against water loss and damage, as well as an interface for gas exchange; the vasculature functions in the transport of water and nutrients, and provides structural support; and the cortex is often involved in photosynthesis and storage. Access to individual cells and tissues of an organ is difficult using conventional dissection techniques, and tissue-specific analysis of gene expression requires the use of molecular markers or laser microdissection (LMD).

Tissue-specific analysis of the maternal regions of developing fruits and seeds can contribute to our understanding of reproductive development in important crop plants. A tissue-specific transcriptome analysis performed on young tomato fruit revealed spatial separation of the transcripts associated with energy metabolism, secondary metabolite biosynthesis, and cuticle formation, as well as potential source–sink relationships within the maternal tissues of the developing fruit (Matas *et al.*, 2011). The epidermis and subepidermis of *Citrus clementine* have also been characterized at the transcriptomic level (Matas *et al.*, 2010). The funiculus, a maternal structure that connects the developing seed to the fruit of the maternal plant, has been profiled in Arabidopsis seed development (Khan *et al.*, 2015), but no molecular studies have been done on the funiculus of its close relative canola, either whole or at the tissue-specific level. As the funiculus is the only structure connecting the maternal plant and the developing seed, identifying the transcriptomic contribution of each tissue type of the funiculus will allow for a better understanding of the mechanisms by which nutrients, water, and signals are transported to the seed throughout development.

Anatomical studies of Arabidopsis (Khan *et al.*, 2015) and canola (Chan and Belmonte, 2013) reveal different developmental patterns in the primary tissue systems of the funiculus. Perhaps the most dramatic difference between the funiculi of both species is that the cortex remains intact throughout the entirety of funiculus development in Arabidopsis (Khan *et al.*, 2015), while the cortex of the canola funiculus breaks down at the globular stage of seed development (Chan and Belmonte, 2013). Cuticular deposition on the outer surface of the epidermal cells is observed in Arabidopsis (Khan *et al.*, 2015), but not in canola. Thickening of the cell wall is also more extensive in Arabidopsis epidermal cells, though the epidermis undergoes cell death in both genera. Comparative anatomical studies have also revealed remarkable differences in funicular structure within the Leguminosae (Endo, 2012). By examining the three tissue systems of the canola funiculus at the transcriptomic level, we can provide insight into the biological function of each tissue, and the roles they play in supporting seed development.

Recently, Khan *et al.* (2015) compared the Arabidopsis funiculus with other seed subregions at the transcript level and found that the funiculus is a transcriptionally distinct structure within seed development. Gene enrichment and anatomical analysis revealed the funiculus to be heavily involved in transport and the processing of macromolecules. Further, we previously found that all three tissue systems (dermal, ground, and vascular) of the canola funiculus undergo dramatic anatomical changes during the globular stage of seed development (Chan and Belmonte, 2013). These changes occur in tandem with tissue patterning and morphogenesis in the canola embryo, which accumulates transcripts associated with auxin response and transcriptional regulation (Venglat *et al.*, 2013). Here, we chose to examine the funiculus at the globular stage of canola seed development to reveal the molecular patterns that contribute to the identity and function of the three tissue systems at one of the earliest stages of seed development.

The purpose of this study is to investigate tissue-specific transcriptional programs underlying funiculus development in the globally important species *Brassica napus* (canola) (Rempel *et al.*, 2014). To better understand tissue system development in the funiculus, we used a combination of LMD coupled to next-generation RNA sequencing (RNA-seq) to provide a high-resolution spatial account of transcript populations within this critical and understudied region of the seed. The current study expands on work previously reported by Belmonte *et al.* (2013) and Khan *et al.* (2014) who profiled mRNAs in every subregion of the embryo, endosperm, and seed coat of the Arabidopsis seed over the course of development.

In the current study, we describe gene activity in the three primary tissues of the funiculus and reveal putative developmental processes underlying the epidermis, cortex, and vasculature, and suggest the integrative roles of these tissues in supporting seed development. Our tissue-specific analysis increases the spatial resolution of the canola funiculus transcriptome—we identified >7700 tissue-specific transcripts that were undetected in whole funiculus (WF) tissues. Our results show that spatial patterns of transcript accumulation are specific to each tissue type. Transcripts associated with cell wall modification accumulate primarily in the epidermis, the funiculus cortex accumulates transcripts associated with growth and gibberellic acid (GA)-mediated signaling, and the vascular tissues accumulate transcripts associated with vascular differentiation and development, secondary cell wall biosynthesis, and transport. We further discuss how these patterns of transcript accumulation contribute to the development of the individual tissues, and how these tissue-specific processes contribute to the function of the funiculus as a whole.

## Materials and methods

### Plant materials and growth

*Brassica napus* (cv. ‘Topaz’, line DH4079) plants were grown in Sunshine Mix #1 (Sun Gro Horticulture, Agawam, MA, USA) under long-day conditions (16h light, 100–150 µmol photons m^–2^ s^–1^) at 22 ºC with 50–70% relative humidity. Open flowers were pollinated and siliques allowed to develop for 7 d; this corresponds to the globular stage of embryo development. Siliques at 7 days after pollination (DAP) were collected and processed as detailed below.

### Laser microdissection (LMD)

#### Tissue processing and embedding

**Fig. 1.**
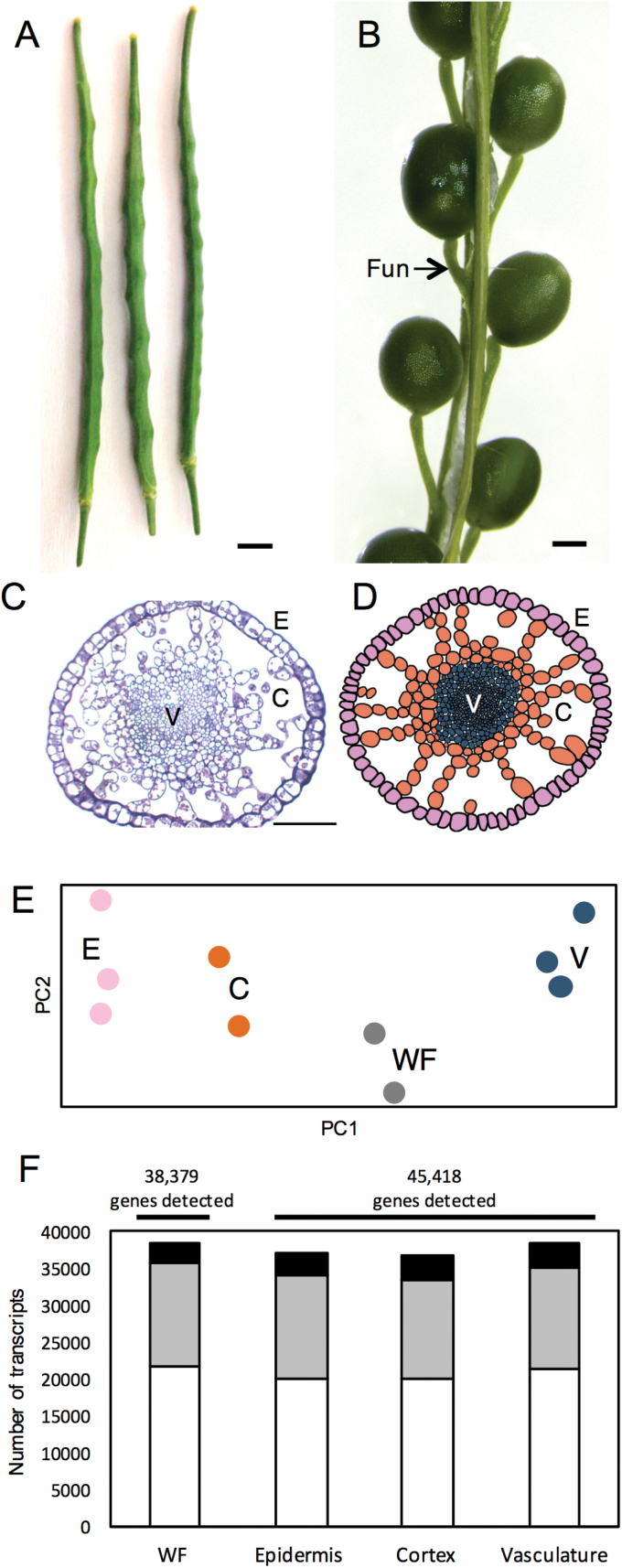
Tissue-specific mRNA profiling of the globular-stage funiculus in *Brassica napus*. (A) Whole siliques, scale bar=0.45mm. (B) Silique with valves removed to reveal seeds and funiculi (Fun); scale bar=0.5mm (C) Cross-section of the funiculus; E, epidermis; C, cortex; V, vasculature; scale bar=100 µm. (D) Schematic diagram of the funiculus showing the three plant tissue systems accessible through LMD: the epidermis (pink), cortex (orange), and vasculature. (E) Principal component analysis of tissue-specific RNA-seq datasets—the epidermis (E, pink), cortex (C, orange), whole funiculus (WF, grey), and vasculature (V, navy blue) all form distinct groups. (F) Distribution of low (white, 1 ≤ FPKM < 5), moderately (gray, 5≤ FKPM < 25), and highly (black, 25 ≤ FPKM) accumulating transcripts identified in the different tissues of the funiculus.

Siliques at the globular stage of embryo development (7 DAP; Fig. 1A, B) were fixed in 25% glacial acetic acid and 63.75% ethanol overnight at 4 ºC. The next day, tissues were rinsed with 70% ethanol (3×), and then dehydrated in a graded ethanol series: 85, 95, and 100% (2×), with the tissues remaining in each solution in the series for ~30min. Tissues were then further dehydrated with xylenes, which also served as the transition solvent. Tissues were placed in a series of graded xylene–ethanol solutions in the following ratios (v/v): 1:3, 1:1, and 3:1, with the tissues remaining in each solution for 1h. The tissues were then transferred to 100% xylene overnight.

Xylene solution was replaced with fresh 100% xylene for 2h. After 2h, six Paraplast Plus paraffin chips (McCormick Scientific, St. Louis, MO, USA) were added to the vial. At the end of the day, another 10 chips were added. Tissues were then incubated at 42 ºC for ~1.5h and then at 60 ºC for ~30min. The xylene–paraffin mixture was replaced with 100% paraffin and infiltration occurred at 60 ºC. At the end of the day, another paraffin change was performed.

Another paraffin change was completed the next day and the tissues were left to incubate at 60 ºC for several hours, before the tissues were embedded in 100% melted paraffin wax at room temperature. The cooled, solidified molds were stored at 4 ºC overnight. All solutions and tools used throughout were RNase free. Unless otherwise specified, tissue processing was completed in a rotary mixer at room temperature.

#### Sectioning, mounting, and de-waxing

Sections 10 µm thick were cut using disposable steel blades on a Leica RM2245 microtome (Leica Microsystems, Wetzlar, Germany) and mounted on MembraneSlides (Leica Microsystems). The sections were later de-waxed through submergence in xylenes for 1min.

#### LMD of tissues

LMD was carried out using the Carl Zeiss PALM MicroBeam system (Carl Zeiss, Oberkochen, Germany). The desired tissue fragment (‘element’) was traced using the Freehand Tool in the PALMRobo software version 4.3. Laser values were specified in such a way as to minimize the amount of energy utilized to minimize tissue damage. These values were set to 15 for speed and ~45 for energy. Epidermal, cortical, and vascular tissues (Fig. 1C, D) were collected in separate 0.5ml microcentrifuge tube caps (Fisher Scientific, Ottawa, ON, Canada) lined with 30 µl of lysis buffer [from the Ambion^®^ RNAqueous^®^-Micro Kit (Life Technologies, Carlsbad, CA, USA)]. WFs were also collected as a control. The collection period for a single cap did not exceed 1.5h. If the lysis buffer did evaporate during this time, 20–30 µl of lysis buffer was added back to the cap and the added volume was noted. Samples that were not used immediately for RNA extractions were stored at –80 ºC.

### RNA isolation, library preparation, and RNA-sequencing

#### RNA extraction and amplification

At least 20 elements from funiculi from at least three different plants were combined to constitute a single biological replicate. Three biological replicates per tissue type as well as the three biological replicates of WFs were processed (Supplementary Table S1 at *JXB* online). RNA was extracted using the Ambion^®^ RNAqueous^®^-Micro Kit (Life Technologies). Samples that were not analyzed immediately were stored at –80 ºC.

The cDNA libraries were synthesized from the RNA samples using the Ovation^®^ RNA-Seq System V2 kit (NuGEN, San Carlos, CA, USA) according to the manufacturer’s instructions. The libraries were fragmented using the NEBNext^®^ dsDNA Fragmentase^®^ kit (New England Biolabs, Ipswich, UK). The cDNA libraries were then further refined using the Illumina TruSeq™ RNA Sample Preparation v2 kit (Illumina, San Diego, CA, USA) using the low-throughput protocol according to the manufacturer’s instructions.

#### Validating sample quality

The extracted RNA samples and the cDNA libraries were loaded into Agilent RNA 6000 Pico Chips (Agilent Technologies, Santa Clara, CA, USA) and Agilent High Sensitivity DNA Chips (Agilent Technologies), respectively, according to the manufacturer’s instructions. The chips were run in an Agilent 2100 Bioanalyzer (Agilent Technologies) according to the manufacturer’s instructions. The electropherogram tracings can be found in Supplementary Table S1. Because the library for Cortex 3 was not suitable for sequencing (Supplementary Table S1), it was excluded from the pool.

#### RNA-sequencing

The Illumina HiSeq 2500 platform was used to sequence the multiplexed samples on a single lane of a flow cell according to the manufacturer’s instructions. Each sample was sequenced at 50bp, single-end reads.

### Analysis of the sequencing data

Fastq files from UC Davis Genome Center (http://genomecenter.ucdavis.edu/) containing 50bp single-end Ilumina reads were mapped to the publically available *B. napus* reference genome downloaded from Genosope (http://www.genoscope.cns.fr/) using TopHat (v2.0.13). A summary of the read alignments can be found in Supplementary Table S2. Transcript abundances were calculated as fragments per killobase of transcript per million mapped reads (FPKMs) using the Cufflinks pipeline (v2.2.1, http://cole-trapnell-lab.github.io/cufflinks/). To avoid contamination of data with transposable elements and tRNAs, transcripts without a translated protein blast hit to *Arabidopsis thaliana* TAIR10 proteins were dropped.

The raw RNA-seq files were deposited in the NCBI Gene Expression Omnibus (GEO) database, accession number: GE71859.

#### Visualization of RNA sequence data

##### Quality assessment and determination of the relationships between biological replicates.

DESeq (differential expression of RNA-seq data at the gene level, version 1.16.0) was used to determine the relationships between biological replicates and tissue types. A transcript was considered to be present at FPKM >1. Because the WF2 biological replicate grouped separately from all other samples (Supplementary Fig. S1), we repeated our analysis without the WF2 data, and all remaining analyses also excluded the WF2 data. All remaining analyses were conducted using the average FPKM values for each tissue.

##### Quantification and visualization of unique transcripts found in individual tissue layers.

A fold-change analysis was carried out using CuffDiff (Trapnell *et al.*, 2012) in order to find genes significantly differentially expressed between tissues. A gene was considered to be tissue enriched if it was statistically differentially expressed (*P*<0.005, *q*<0.05) and expressed at levels at least 5-fold higher in one tissue relative to the other two tissues. A heatmap of the tissue-enriched transcripts and their associated non-scaled FPKM values was created using gplots in R (Studio version 0.98).

##### Gene Ontology (GO) term enrichment.

Enrichment of the genes associated with each tissue was carried out using CanEnrich, a program modified from ChipEnrich (Orlando *et al.*, 2009) for enrichment in *B. napus*. A GO term was considered enriched if the log_10_ *P*-value was less than or equal to –3 (i.e. *P*≤0.001). In order to inform on the regulation of biological processes in each tissue, we also generated transcriptional modules. These modules were visualized using Cytoscape (http://www.cytoscape.org).

##### Transmission electron microscopy (TEM).

Tissue processing, embedding, blocking, sectioning, visualization, and image modifications were performed exactly as described in Chan and Belmonte (2013).

## Results

### The three tissue types of the funiculus are transcriptionally distinct

To determine the relationship between the transcript populations of each tissue type, we performed a principal component analysis (PCA), which shows that the biological replicates of each tissue type cluster together (Fig. 1E; Supplementary Fig. S1). Each tissue type formed a distinct group, separate from the WF tissue samples, indicating unique transcriptional programs in each tissue. The vasculature (V) mRNA populations were the most distinct, grouping furthest from the cortex (C) and epidermal (E) transcriptome populations.

### Similar numbers of genes are found in all funiculus tissue samples

RNA-seq of the funicular tissues revealed a total of 46 140 detected transcripts (FPKM ≥1; Supplementary Dataset S1) representing ~45.7% of the predicted canola gene models (Chalhoub *et al.*, 2014). The total number of transcripts detected was similar for all tissues examined: an average of 37 084 transcripts were detected in the epidermis, 36 695 in the cortex, 38 368 in the vasculature, and 38 379 in the WF (Supplementary Table S3). We then divided the transcripts into those with low, moderate, and high abundance. Similar numbers of low (1 ≤ FPKM ≤ 5; ~53.7–56.2% of the total number of detected transcripts), moderate (5 < FKPM ≤ 25; ~36.4–37.8% of the total number of detected transcripts), and high (FPKM > 25; ~7.3–8.8% of the total number of detected transcripts) transcript abundance were detected in all samples (Fig. 1F; Supplementary Table S3).

Sequencing of WF samples uncovered 38 379 transcripts (FPKM ≥1), but, when the individual tissues collected using LMD were compared with the WF transcriptome population, we were able to detect an additional 7761transcripts (Fig. 1F); this accounts for 16.8% of all transcripts detected. Thus, using LMD to capture individual tissues of the funiculus improves the number of transcripts detected compared with WF samples.

### The epidermis, cortex, and vasculature transcriptomes each contain unique sets of transcripts

**Fig. 2.**
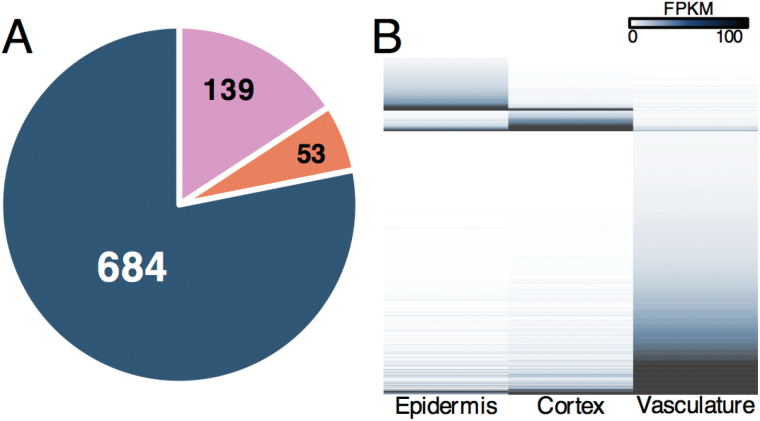
Transcript specificity in LMD-acquired tissues from the globular-stage canola (*Brassica napus*) funiculus. (A) Pie chart showing the number of tissue-specific transcripts in the epidermis (pink), cortex (orange), and vasculature (navy blue) of the funiculus. Transcripts were considered to be specific if they were significantly differentially expressed at a level at least 5-fold higher in one tissue relative to the other tissues. (B) Heatmap showing accumulation of tissue-specific transcripts.

A fold-change analysis was carried out to identify differentially expressed transcripts (Supplementary Dataset S1). A transcript was considered enriched in a tissue if it was significantly differentially expressed at levels at least 5-fold higher relative to the other two tissues (*P*<0.005, *q*<0.05). A total of 876 genes were found to be tissue enriched (Fig. 2A). The highest number of enriched transcripts accumulated in the vasculature, where we identified 684 enriched transcripts (Fig. 2A). Data revealed 139 enriched transcripts accumulated in the epidermis, and 53 enriched transcripts in the cortex, the fewest of the three tissue systems (Fig. 2A). Visualization of gene expression confirms the tissue-dependent accumulation of these transcripts (Fig. 2B)

### GO enrichment analysis reveals biological processes enriched in the epidermis, cortex, and vasculature of the funiculus

**Fig. 3.**
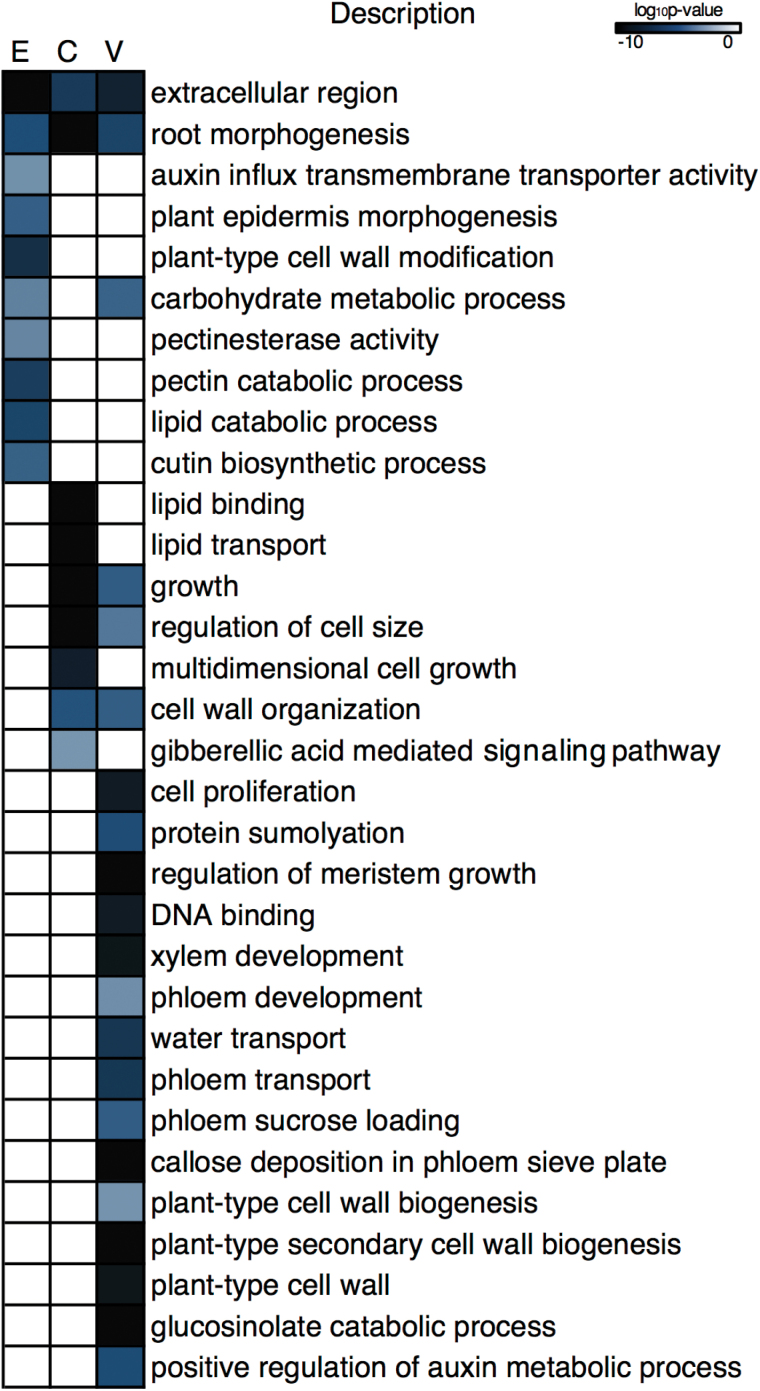
Heatmap of select Gene Ontology terms enriched in tissue-specific transcript populations, highlighting tissue-specific biological processes operative within the globular-stage canola funiculus.

We performed a GO enrichment analysis of each tissue-specific gene list to provide insight into the biological processes putatively underlying each tissue system of the funiculus (Fig. 3). The complete list of GO terms (with their log_10_ *P*-values) associated with each tissue, as well as the list of genes belonging to each GO term can be found in Supplementary Dataset S2.

### The epidermis of the funiculus is involved in cell wall modification and epidermal morphogenesis

**Fig. 4.**
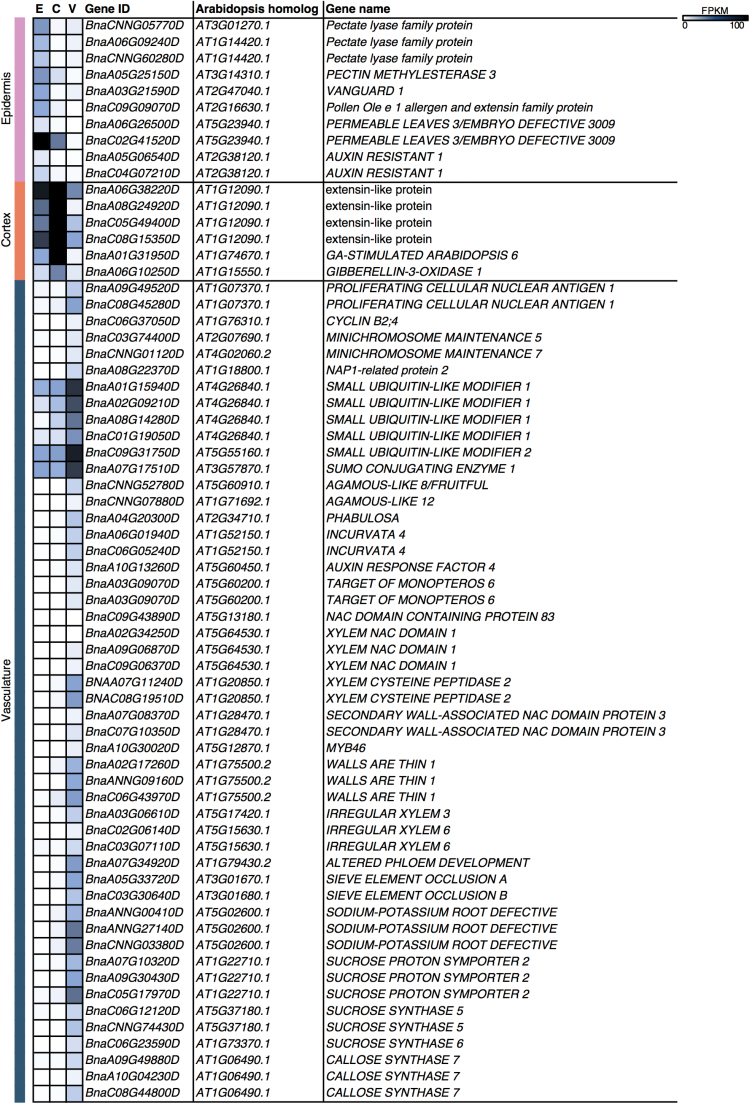
Heatmaps of the FPKMs of select tissue-specific genes identified in the *B. napus* funiculus.

The population of transcripts enriched in the epidermis (Fig. 2A; Supplementary Dataset S1) were enriched for plant epidermis morphogenesis (*P*=2.86 E-5), cutin biosynthesis (*P*=3.28 E-5), plant-type cell wall modification (*P*=4.69 E-8), and pectin catabolism (5.49 E-7) (Fig. 3). Several genes encoding pectate lyase family proteins (*BnaCNNG05770D*, *BnaA06G09240D*, and *BnaCNNG60280D*) were found within these GO terms, as well as one homolog of *PECTIN METHYLESTERASE 3* (*PME3*; *BnaA05G25150D*) and the pectin methylesterase *VANGUARD 1* (*VGD1*; *BnaA03G21590D*) (Fig. 4). A gene encoding an extensin family protein (*BnaC09G09070D*) was also identified. In addition, two *B. napus* homologs of the acyl transferase *PERMEABLE LEAVES 1* (*PEL3*; *BnaA06G26500D* and *BnaC02G41520D*) were identified in the plant epidermis morphogenesis and cutin biosynthesis GO terms (Fig. 4).

**Fig. 5.**
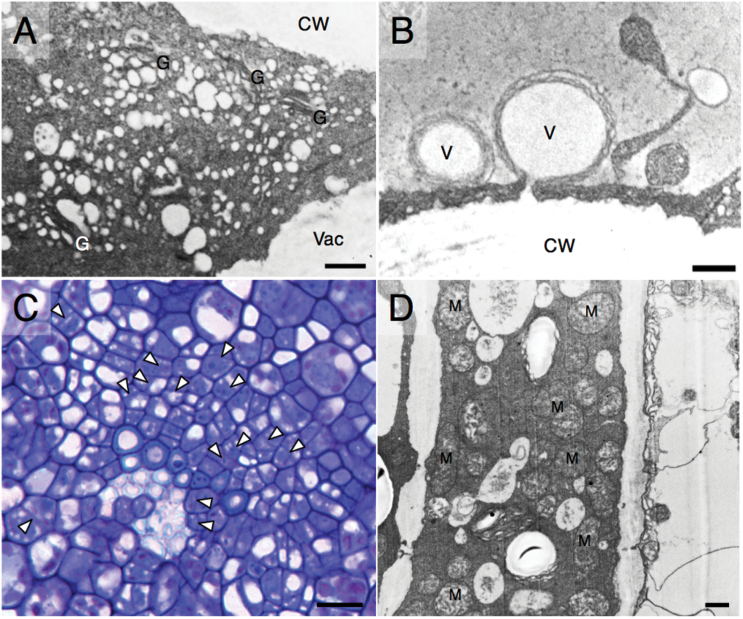
Micrographs highlighting anatomical features of the *B. napus* funiculus. (A) Transmission electron micrograph of a characteristic epidermal cell showing the outer tangential cell wall (CW), the vacuole (Vac), and numerous Golgi apparatuses (G); scale bar=500nm. (B) Transmission electron micrograph showing fusion of vesicles (V) with the cell wall (CW); scale bar=500nm. (C) Light micrograph of the vascular strand of the funiculus. Recently divided cells are indicated with arrowheads; scale bar=12.5 µm. (D) Transmission electron micrograph showing numerous mitochondria (m) present in the non-conducting cells of the phloem; scale bar=500nm.

Validating our enrichment analysis, TEM images show numerous Golgi complexes and vesicles located proximal to the outer tangential cell wall of epidermal cells (Fig. 5A). We also identified extensive vesicular fusion with the epidermal cell walls (Fig. 5B), which suggests that the function of Golgi activity in the epidermis of globular-stage funiculi contributes to the delivery of materials and enzymes to modify the epidermal cell walls at the globular stage of development. Two homologs of *AUXIN RESISTANT 1* (*AUX1*; *BnaA05G06540D* and *BnaC04G07210D*) (Fig. 4), which has been shown to associate with active vesicles (Kleine-Vehn *et al.*, 2006), were identified in the root morphogenesis GO term, indicating a potential role for hormonal signaling in the regulation of the development of the funiculus epidermis.

### Cell growth is associated with cortex development in the *B. napus* funiculus

Cortex-enriched transcripts were enriched for growth (*P*=3.77 E-11), regulation of cell size (*P*=3.89 E-11), and GA-mediated signaling (9.64 E-4) (Fig. 3). Four homologs of a gene encoding an extensin-like protein (*BnaA06G38220D*, *BnaA08G24920D*, *BnaC05G49400D*, and *BnaC08G15350D*) (Fig. 4) were identified in the growth and regulation of cell size GO terms. Additionally, *GA-STIMULATED ARABIDOPSIS 6* (*GASA6*; *BnaA01G31950D*), and *GIBBERELLIN-3-OXIDASE 1* (*GA3OX1*; *BnaA06G10250D*), which is involved in GA biosynthesis (Fig. 4), were found in the GA-mediated signaling pathway GO term. The tissue-specific accumulation of these transcripts may suggest that cell development in the cortex of the globular-stage funiculus relies heavily on processes of cell growth and expansion.

### The vasculature of the funiculus is metabolically active and accumulates transcripts associated with vascular development, secondary cell wall biosynthesis, transport, and hormone response

Vasculature-enriched transcripts were enriched for processes such as cell proliferation (*P*=1.28 E-9) and protein sumolyation (*P*=7.69 E-6) (Fig. 3). Genes associated with cell cycle regulation and chromosome maintenance were found in the cell proliferation GO term, including two homologs of *PROLIFERATING CELLULAR NUCLEAR ANTIGEN 1* (*PCNA1*; *BnaA09G49520D* and *BnaC08G45280D*), *CYCLIN B2;4* (*CYCB2;4*; *BnaC06G37050D*), *MINICHROMOSOME MAINTENANCE 5* (*MCM5*; *BNAC03G74400D*), *MINICHROMOSOME MAINTENANCE 7* (*MCM7*; *BnaCNNG01120D*), and *NAP1-RELATED PROTEIN 2* (*NRP2*; *BnaA08G22370D*) (Fig. 4). Four homologs of *SMALL UBIQUITIN-LIKE MODIFIER 1* (*SUMO1*; *BnaA01G15940D*, *BnaA02G09210D*, *BnaA08G14280D*, and *BnaC01G19050D*) were identified in the protein sumolyation GO term, as well as *SMALL UBIQUITIN-LIKE MODIFIER 2* (*SUMO2*; *BnaC09G31750D*) and *SUMO CONJUGATING ENZYME 1* (*SCE1*; *BnaA07G17510D*). Anatomical analysis shows many cells in the vascular strand that have recently undergone cell division (Fig. 5C). These data indicate that the vascular strand of the globular stage canola funiculus is actively involved in growth via cell proliferation.

**Fig. 6.**
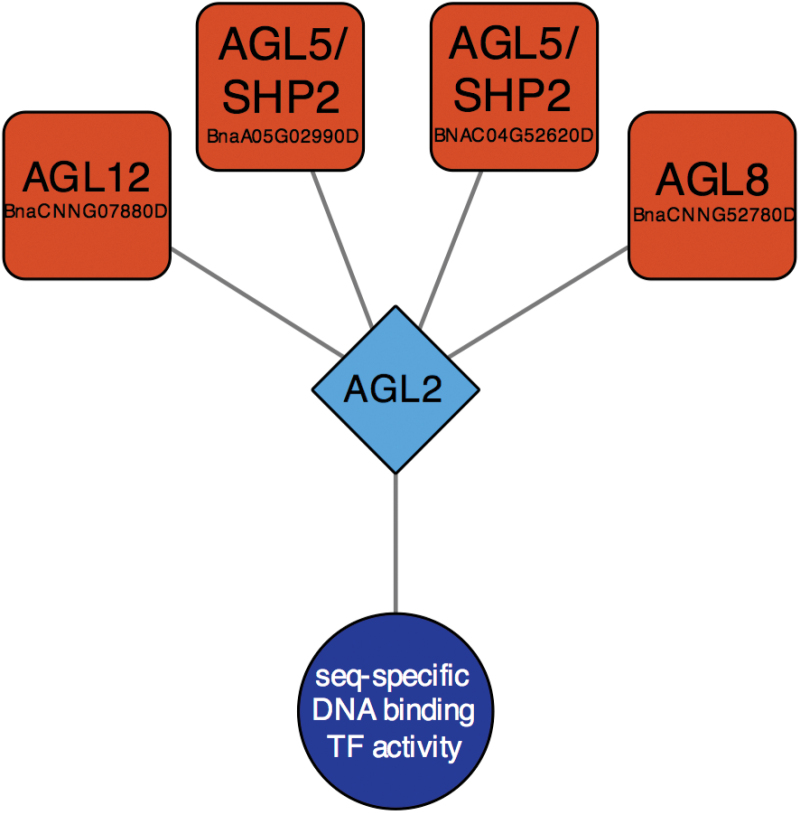
Predictive regulatory network identified in the population of vascular-specific transcripts. Transcription factors are indicated with orange oblongs, DNA-binding motifs with light blue diamonds, and GO terms with dark blue circles.

Both xylem (*P*=3.22 E-10) and phloem development (*P*=6.39 E-4) were enriched in the vasculature-enriched transcripts (Fig. 3). DNA binding (*P*=1.20 E-9) was enriched in the vasculature; transcripts encoding MADS box transcription factors, *AGAMOUS-LIKE 8* (*AGL8*; *BnaCNNG52780D*) and *AGAMOUS-LIKE 12* (*AGL12*; *BnaCNNG07880D*), as well as a HD-Zip transcription factor *PHABULOSA* (*PHB*; *BnaA04G20300D*), and other transcription factors including *AUXIN RESPONSE FACTOR 4* (*ARF4*; *BnaA10G13260D*), and two homologs of *TARGET OF MONOPTEROS 6* (*TMO6*; *BnaA03G09070D* and *BnaA03G09070D*) (Fig. 4) were identified in the DNA binding GO term. Using further enrichment analyses to identify DNA-binding motifs and transcriptional regulators within the populations of tissue-enriched transcripts, we identified a transcriptional network that predicts several MADS box transcription factors—AGL5/SHP2 (*BnaA05G02990D* and *BnaC04G52620D*), AGL8 (*BnaCNNG52780D*), and AGL12 (*BnaCNNG07880D*)—as regulators of DNA binding activity in the vasculature of the globular-stage canola funiculus (Fig. 6).

Several negative regulators of vascular differentiation were identified in the xylem development GO term, including two homologs of the HD ZIP III transcription factor *INCURVATA 4* (*ICU4*; *BnaA06G01940D* and *BnaC06G05240D*), *NAC DOMAIN CONTAINING PROTEIN 83* (*NAC083*; *BnaC09G43890D*), three homologs of *XYLEM NAC DOMAIN 1* (*XND1*; *BnaA02G34250D*, *BnaA09G06870D*, and *BnaC09G06370D*) (Fig. 4), and two homologs of *SECONDARY WALL-ASSOCIATED NAC DOMAIN PROTEIN 3* (*SND3*; *BnaA07G08370D* and *BnaC07G10350D*). *MYB46* (*BnaA10G30020D*), a target of *SECONDARY WALL-ASSOCIATED NAC DOMAIN PROTEIN 3*, was found in the secondary cell wall biogenesis GO term (*P*=2.09 E-11). Other transcripts associated with secondary cell wall thickening were identified, including *WALLS ARE THIN 1* (*WAT1*; *BnaA02G17260D*, *BnaANNG09160D*, and *BnaC06G43970D*), *IRREGULAR XYLEM 3* (*IRX3*; *BnaA03G06610D*), and *IRREGULAR XYLEM 6* (*IRX6*; *BnaC02G06140D* and *BnaC03G07110D*) (Fig. 4). The light-blue staining of the xylem elements in the globular stage funiculus indicates that secondary cell wall deposition has already begun at this stage of development (Fig. 5C). Two homologs of *XYLEM CYSTEINE PEPTIDASE 2* (*XCP2*; *BnaA07G11240D* and *BnaC08G19510D*), involved in vessel element autolysis (Avci *et al.*, 2008), were identified in the regulation of meristem growth GO term (*P*=2.14 E-14). Therefore, key genes involved in xylem formation and development are co-expressed in the vasculature of the globular-stage canola funiculus.

The vasculature-specific transcript population was also enriched for phloem development (*P*=6.39 E-4); *ALTERED PHLOEM DEVELOPMENT* (*APL*; *BnaA07G34920D*), *SIEVE ELEMENT OCCLUSION A* (*SEOA*; *BnaA05G33720D*), and SIEVE ELEMENT OCCLUSION B (*SEOB*; *BnaC03G30640D*) (Fig. 4) were found in the phloem development GO term. The vasculature was also enriched for water transport (*P*=1.58 E-7), phloem transport (*P*=1.94 E-7), phloem sucrose loading (*P*=2.61 E-5), and callose deposition in the phloem sieve plate (*P*=9.15 E-11). Transcripts associated with phloem transport and function were found within the phloem transport and phloem sucrose loading GO terms, including three homologs of *SODIUM-POTASSIUM ROOT DEFECTIVE 1* (*NAKR1*; *BnaANNG00410D*, *BnaANNG27140D*, and *BnaCNNG03380D*), and three homologs of *SUCROSE PROTON SYMPORTER 2* (*SUT2*; *BnaA07G10320D*, *BnaA09G30430D*, and *BnaC05G17970D*) (Fig. 4). *SUCROSE SYNTHASE 5* (*SUS5*; *BnaC06G12120D* and *BnaCNNG74430D*), *SUCROSE SYNTHASE 6* (*BnaC06G23590D*), and *CALLOSE SYNTHASE 7* (*CALS7*; *BnaA09G49880D*, *BnaA10G04230D*, and BnaC08G44800D) (Fig. 4) were within the callose deposition in phloem sieve plate GO term. Anatomical analysis reveals numerous densely packed mitochondria in the cells of the phloem (Fig. 5D), suggesting that phloem development and function are energy-demanding processes in the funiculus. Therefore, the globular-stage funiculus vasculature is enriched for the regulation of the development and function of both xylem and phloem.

## Discussion

### Dissecting individual tissues of the funiculus increases the resolution of the funiculus transcriptome

LMD coupled with next-generation RNA-seq provides valuable insight into the spatial distribution of gene activity in complex multicellular biological systems such as the canola funiculus. Khan *et al.* (2015) reported that the Arabidopsis funiculus contains unique transcripts compared with the rest of the seed regions and subregions. In the present study, we extended our work to the funiculus of the valuable oilseed *B. napus* at the tissue-specific level. Combining LMD with bioinformatics approaches, we were able to detect unique transcripts present among the tissue systems of the canola funiculus (Fig. 2A, B; Supplementary Dataset S1). Although the total number of transcripts was similar in all dissected tissue, as well as the whole funiculus, >16% of all transcripts detected are tissue enriched (Fig. 2B) and would not have been revealed without analyzing these tissues individually. This increased resolution of the funiculus transcriptome allows us to focus more in depth on the biological processes occurring within the funiculus during seed development. Anatomical evidence provides support for the enrichment analyses of the transcriptome data, and reveals the dynamic nature of each tissue system in the *B. napus* funiculus. Overall, these data provide novel insights into the transcriptomic patterns that operate in each tissue of the funiculus that contribute to its function in supporting seed development.

### Tissue systems of the canola funiculus are transcriptomically distinct

Each of the tissue systems contain unique populations of transcripts (Figs 1E, 2C), suggesting distinct biological processes and specific roles for each of the three tissue systems in funiculus and seed development. Both the funiculus vasculature and epidermis mRNA populations contained greater numbers of tissue-enriched transcripts than the paranchymatous cortex. A similar pattern of transcript accumulation has been observed in maternal reproductive tissues of tomato (Matas *et al.*, 2011), reflective of the unique and dramatic changes that both tissue types undergo in plant fruit structures. While each region of the funiculus is transcriptionally distinct, the transcriptomic profiles of the epidermis and cortex are more similar, and the vasculature profile is more distinct from that of the other tissues. Transcriptome studies have found large differences between the mRNA populations of the epidermis and vasculature (Nakazono *et al.*, 2003) in maize coleoptiles, as well as between the mesophyll and vasculature of maize leaves (Chang *et al.*, 2012). The vasculature of the *B. napus* funiculus may have a more unique population of transcripts as it contains more diverse cell types than the epidermis or cortex, which are both relatively uniform in cell type (Chan and Belmonte, 2013).

#### The epidermis is associated with cell wall modification, resulting in a resistant structure that supports the maturing funiculus

During early funiculus development, the cells of the epidermis undergo cell enlargement and cell wall biosynthesis, with cell wall thickening becoming evident at the globular stage of development (Chan and Belmonte, 2013). We hypothesize that at the globular stage of embryo development, the funiculus is at the beginning of a transition from cell growth to cell wall thickening in the epidermal tissue. In support of this, we found that transcripts enriched in the epidermis were enriched for biological processes that are related to cell wall modification and pectin metabolism (Fig. 3). Our anatomical analysis reveals the presence of numerous Golgi apparatuses adjacent to the distal cell wall of the epidermal cells of the funiculus, as well as the fusion of vesicles with the cell wall; these Golgi may participate in providing carbohydrate substrates for cell wall development and thickening. Interestingly, while the Arabidopsis funiculus epidermis accumulates a thick cuticle throughout its development, and is transcriptomically enriched for wax and cuticle biosynthesis at the globular stage of seed development (Khan *et al.*, 2015), the canola funiculus lacks prominent cuticular deposition, and is enriched only for cell wall development.

Two pectin methylesterases—PME3 and VGD1—are predicted to be involved in pectin catabolism in the funiculus epidermis. The modification of pectins by pectin methylesterases has been shown to correspond to increased cell wall rigidity, thus limiting the growth of the cell (reviewed in Wolf and Greiner, 2012). The pectin methylesterase VGD1 has been implicated in the maintenance of cell wall integrity in pollen tube growth growth (Jiang *et al.*, 2005). Increased cell wall rigidity may contribute to the structural integrity of the funiculus epidermis, which is hypothesized to undergo programmed cell death over the course of canola funiculus development (Chan and Belmonte, 2013), and may contribute to the strength of the funiculus organ itself. Furthermore, the development of the epidermis may have consequences for the determination of organ size through signaling (Savaldi-Goldstein *et al.*, 2007) and structural limitation. It is not yet known how genes associated with cell wall modification are involved in the regulation of epidermal cell growth and the delimitation of organ size of the funiculus in canola.

#### Gibberellic acid-mediated signaling may be associated with growth and cell expansion in the cortex

We identified GASA6 as a cortex-specific transcript associated with GA response and signaling in the funiculus. The cortex of the funiculus undergoes rapid cell growth and proliferation post-fertilization, remaining functional during the globular stage of seed development but undergoing degradation by the end of morphogenesis (Chan and Belmonte, 2013). This is in contrast to Arabidopsis, where the cortex of the funiculus remains intact at seed maturity (Khan *et al.*, 2015). We found that four homologs of a gene encoding an extensin-like protein were also expressed at levels at least 5-fold higher in the cortex relative to the vascular and epidermal tissues of the funiculus. Extensins have been shown to be essential to cell division during embryo development (Hall and Cannon, 2002). Zhong *et al.* (2015) suggest that GASA6 may act to co-ordinate GA signaling with cell growth in the embryo axis during seed germination, and so it may be possible that it could be performing a similar function of integrating GA signaling and cell growth in the cortex of the funiculus. GASA6 is predicted to act downstream of RGA-LIKE 2 (RGL2) (Zhong *et al.*, 2015) in Arabidopsis. *RGL2* is expressed in the funiculus and chalazal seed coat of morphogenesis-stage Arabidopsis seeds, suggesting a potential role in GA-mediated signaling, though its role in the funiculus tissue is unknown. We did not find *RGL2* to be expressed in the *B. napus* funiculus, though two homologs of *RGA-LIKE 1* were expressed non-specifically in the cortex. Given that the cortex of the Arabidopsis and canola funiculi undergo distinctly different developmental fates throughout seed development, it is possible that distinct transcriptional programs are operating in the funiculus cortexes of these species. Taken together, the role of GA-mediated signaling in the cortex of the funiculus remains uncharacterized in any plant species, though our data support that GA-mediated signaling may be integrated with cell expansion and growth in the cortex of the globular-stage funiculus.

#### The vasculature of the globular-stage canola funiculus is an actively proliferating tissue whose development may be regulated by several AGAMOUS-like transcription factors

Our enrichment analysis revealed that the vasculature is heavily enriched for cell proliferation. The tissue-specific expression of *PCNA1*, and *MCM5* and *7*, which are expressed preferentially in actively proliferating cells and involved in DNA synthesis during replication (Shultz *et al.*, 2009; Strzalka and Ziemienowicz, 2010), indicates that the vasculature is more heavily involved in processes of cell division than the cortex or epidermis at the globular stage of seed development. While the vascular strand continues to enlarge throughout funiculus development, the most dramatic increase in size results from cell proliferation that occurs in the time period between fertilization and early morphogenesis (Chan and Belmonte, 2013). This active cell division would require strict cell cycle control in order to ensure proper development of the vascular strand. Our data suggest that post-translational modification via protein sumoylation may be a key factor in the regulation of cell cycle control in the funiculus vasculature. PCNA1 undergoes sumoylation via the action of several SUMO proteins in vitro, including SUMO1 (Strzalka *et al.*, 2012). Both *PCNA1* and *SUMO1* are co-expressed with *SCE1*, a key regulator of the cell cycle and meristematic proliferation, in the globular-stage canola funiculus.

Very little is known about the regulation of the cell cycle and growth in the funiculus in any plant species; however, our enrichment analyses identified AGL8, AGL12, and AGL5 as putative regulators of DNA binding in the vasculature of the funiculus. AGL12 and AGL8 are both regulators of cell proliferation and expansion, and AGL8 is required for proper vascular development (Gu *et al.*, 1998; Tapia-López *et al.*, 2008). AGL5 is implicated in interactions with other MADS-box transcription factors as well as the AP-2-like transcription factor, AINTEGUMENTA (ANT), in order to control cell expansion and division in the Arabidopsis funiculus. How these AGAMOUS-like transcription factors, cell cycle regulators, and regulators of protein sumoylation work together to co-ordinate vascular development in the funiculus is unknown, but understanding how vascular proliferation contributes to the structure of the canola funiculus, and the consequences this growth has for the ability of the funiculus to transport nutrients to the developing seed, will be valuable in our understanding of canola seed development overall.

#### Competition at the transcriptional level between xylem and phloem proliferation in the vascular strand may influence the structure and function of the canola funiculus

Despite the importance of the vasculature of the funiculus in delivering nutrients from the maternal plant to the developing seed, the regulation of vascular development in the funiculus is generally not well characterized, and little to no information about the development of the vascular strand in the funiculus is available, particularly in *B. napus* studies. Our tissue-specific transcriptomic analysis sheds light on the potential mechanisms that control funiculus development.

Two HD-ZIP III transcription factors, PHB and ICU4, were identified as vasculature-specific transcripts potentially involved in the regulation of vascular development in the funiculus. ICU4 is expressed only in vascular tissues, and is a negative regulator of xylem development (Kim *et al.*, 2005), and PHB regulates vascular patterning (Emery *et al.*, 2003). These developmental regulators are co-expressed in the canola funiculus with *VND-INTERACTING 2* (*VNI2*), which encodes a NAC domain transcription factor that acts to repress xylem vessel formation (Yamaguchi *et al.*, 2010), and *APL*, which represses xylem and promotes phloem development (Bonke *et al.*, 2003). Taken together, these data suggest that there is regulation of cell differentiation and development in the vascular strand, resulting in a greater proportion of phloem relative to xylem at the globular stage of seed development. Early in canola funiculus development, the phloem makes up a greater proportion of the vasculature than xylem, and the two tissues are roughly equal in abundance during the maturation stages of seed development (Chan and Belmonte, 2013). It is possible that the greater proportion of phloem early in seed development allows for the transfer of nutrients, such as precursors to lipids and storage proteins, to the developing seed, and the increase in xylem later in development allows more water transport to support cell expansion and growth of the embryo. Therefore, further examination of the funiculus vasculature at later stages in development is required to understand the transcriptional mechanisms behind the regulation of cell identity in the vascular strand of the canola funiculus that may contribute to seed filling and ultimately seed size.

#### Transcripts associated with vascular development, structure, and function accumulate in the vasculature of the funiculus at the globular stage

Secondary cell wall thickening followed by autolysis is characteristic of xylem vessel development, and contributes its function in water transport. Several genes associated with secondary cell wall thickening in xylem development are co-expressed in the globular-stage funiculus vasculature, including *SND3*, which is specific to the vascular strand of the funiculus, *SND1* (Zhong *et al.*, 2006), and its target MYB46 (Zhong *et al.*, 2007). IRX3, a direct target of MYB46 (Kim *et al.*, 2014), was found to be specific to the vasculature in the funiculus, and IRX6, which is up-regulated in *SND1* overexpression backgrounds (Brown *et al.*, 2005), is also co-expressed in the globular-stage funiculus vasculature. Examination of the funiculus vasculature transcriptome prior to vascular proliferation (during ovule development), as well as at later stages of seed development is required to better understand the regulators of xylem development in the funiculus.

Our tissue-specific analysis of the canola funiculus also sheds light on phloem development in the vascular strand. *SUS5* and *6* are co-expressed with *CALS7* in the vasculature, which is consistent with previous literature that shows exclusive co-expression of these genes in phloem cells (Barratt *et al.*, 2011). It has been suggested that proper callose deposition is integral to the long-distance transport function of the phloem (Barratt *et al.*, 2011), and therefore these sucrose and callose synthase genes may perform an important role in ensuring proper nutrient transfer to the developing seed. *NAKR1* and *SUT2*, key genes involved in the loading of sucrose into phloem cells (Gould *et al.*, 2012) and long-distance source to sink transport of sucrose (Tian *et al.*, 2010), were also co-expressed in the vasculature at the globular stage of development. The phloem cells are densely packed with mitochondria at this stage, which may serve to support the metabolic demands of phloem function and development, as well as provide energy for the rapid proliferation that the phloem tissue undergoes early in seed development (Chan and Belmonte, 2013).

### Conclusions

Tissue-specific transcriptome analysis of the *B. napus* funiculus increases the resolution of the RNA-seq dataset through the identification of genes associated with biological processes of the three primary tissue systems of the plant. We identified biological processes occurring in each tissue system of the funiculus. For example, the epidermis is involved in cell wall development and modification, the cortex is enriched for GA signaling and growth, and the vasculature is heavily involved in the processes of cell division and differentiation. We identify genes and gene regulators putatively involved in these processes, providing insight into the transcriptional circuits that underlie the specialized functions and co-ordinated development of the seed. These data now serve as an important resource for those interested in seed development and targeted manipulation of biological processes for seed improvement research.

## Supplementary data

Supplementary data are available at *JXB* online.

Dataset S1. List of all detected transcripts (mean FPKM ≥1) in the canola (*Brassica napus*) funiculus versus the list of transcripts found in the epidermis, cortex, and/or vasculature biological replicates that were not expressed in the WF biological replicates. Lists of tissue-specific genes (minimum 5-fold significantly differentially expressed in one tissue relative to the other two tissues) and their FPKMs are also presented.

Dataset S2. List of GO terms enriched in tissue-specific genes of the canola funiculus. The log_10_ *P*-values, and list of genes belonging to each GO term is also presented. Genes assigned to enriched GO terms are also presented for each tissue (epidermis, cortex, and vasculature).

Figure S1. Principal component analysis of *B. napus* tissues collected using laser microdissection and profiled using RNA sequencing.

Table S1. Data output including RNA quality, RNA and cDNA quality and quantity, and electropherogram tracings from the Agilent 2100 Bioanalyzer of LMD-collected canola (*Brassica napus*) funiculus epidermis, cortex, vasculature, and whole funiculus.

Table S2. Summary of alignment of RNA-Seq reads to the *B. napus* genome

Table S3. Distribution of low (1 ≤ FPKM < 5), moderate (5≤ FKPM < 25), and high (25 ≤ FPKM) transcripts found in the different tissues of the funiculus.

Supplementary Data

## Abbreviations:

DAP: days after pollination
DESeq: differential expression of RNA-seq data at the gene level
FPKM: fragments per kilobase of exon per million mapped reads
GA: gibberellic acid
GO: Gene Ontology
LMD: laser microdissection
PCA: principal component analysis
RNA-seq: RNA sequencing
TEM: transmission electron microscropy/micrograph
WF: whole funiculus.
