# Supplementary material for: Tissue-specific laser microdissection of the Brassica napus funiculus improves gene discovery and spatial identification of biological processes
Source: J Exp Bot. 2016 May 18;67(11):3561–71. doi: 10.1093/jxb/erw179 (PMC4892738; doi:10.1093/jxb/erw179)
Supplement: Supplementary Data [file supp_67_11_3561__index.html]

Tissue-specific laser microdissection of the Brassica napus funiculus improves gene discovery and spatial identification of biological processes — Tissue-specific laser microdissection of the Brassica napus funiculus improves gene discovery and spatial identification of biological processes — Supplementary Data 

# Tissue-specific laser microdissection of the *Brassica napus* funiculus improves gene discovery and spatial identification of biological processes

## Supplementary Data

Data files

- supplementary\_figure\_S1.pdf - Supplementary Data
- supplementary\_tables\_S1\_S3.xlsx - Supplementary Data
- supplementary\_dataset\_S1.xlsx - Supplementary Data
- supplementary\_dataset\_S2.xlsx - Supplementary Data
