# Supplementary material for: Tissue-specific laser microdissection of the Brassica napus funiculus improves gene discovery and spatial identification of biological processes
Source: J Exp Bot. 2016 May 18;67(11):3561–71. doi: 10.1093/jxb/erw179 (PMC4892738; doi:10.1093/jxb/erw179)
Supplement: Supplementary Data [file supp_erw179_supplementary_figure_S1.pdf]

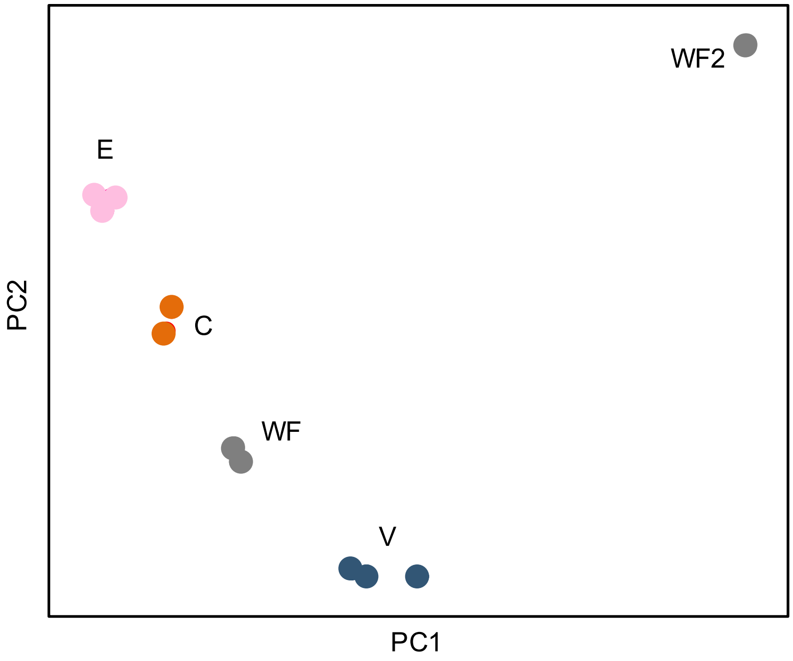

Figure S1: Principal component analysis of *B. napus* tissues collected using laser microdissection and profiled using RNA sequencing. Biological replicates group together and separately from other tissue types. WF2 is an exception and does not group with any of the other samples, therefore, it was excluded from further analyses. E = epidermis; C = cortex; V = vasculature; WF = whole funiculus.
